# Towards personalization of 1D blood flow models: sensitivity analysis and parameter calibration

**DOI:** 10.1007/s10237-026-02094-2

**Published:** 2026-06-10

**Authors:** L. A. Mansilla Alvarez, D. de Oliveira Mussolin, G. Cunha-Lima, S. Garzon, L. O. Müller, P. A. Lemos, P. J. Blanco

**Affiliations:** 1 National Laboratory for Scientific Computing, Petrópolis, Brazil; 2 Hospital Israelita Albert Einstein, São Paulo, Brazil; 3 Department of Mathematics, University of Trento, Trento, Italy; 4 Heart Institute, University of São Paulo Medical School, São Paulo, Brazil; 5 Universitá degli Studi di Roma “La Sapienza”, Rome, Italy; 6 Faculty of Health Sciences, Universidad Autónoma de Chile, Providencia, Chile; 7 Universidade Católica de Petrópolis, Petrópolis, Brazil

**Keywords:** Patient-specific modeling, Computational hemodynamics, Parameter estimation, Global Sensitivity Analysis, CMA-ES

## Abstract

In this work, we address one of the central challenges in translational computational hemodynamics: how to effectively individualize cardiovascular models in scenarios where patient data are scarce and the parameter space is large. We propose an approach to construct patient-specific 1D blood flow models from patient data; specifically, the methodology relies on calibrating model parameters using a limited set of flow rate and pressure measurements. As a model to describe the systemic circulation, we adopt a simplified version of the Anatomically Detailed Arterial Network model which is combined with a Covariance Matrix Adaptation Evolutionary Strategy (CMA-ES) to find the best set of parameters that minimize the discrepancy metric between model prediction and available measurements. The calibration problem is addressed in two steps. In the first one, the influence of each physical parameter on the personalization process is quantified via an exhaustive global sensitivity analysis. Then, CMA-ES is employed to estimate the optimal model parameters. The findings of this study demonstrate the feasibility of deriving accurate patient-specific models in a setting characterized by small data and a high-dimensional parameter space. Furthermore, we provide a comprehensive analysis of the regional impact of physical parameters on the waveform characterization and their potential impact on clinically relevant biomarkers.

## Introduction

In recent decades, computational models have emerged as a feasible tool for describing, analyzing, and predicting physical phenomena that lay beyond the reach of *in-vivo* experimentation, across many fields of biomedical engineering research. In the specific domain of computational hemodynamics, significant efforts have been devoted to verify numerical approximations against both *in-vivo* and *in-vitro* measurements, to relate blood-derived quantities to the onset and progression of cardiovascular diseases, and to provide a robust *in-silico* environment for experimentation. These advances have positioned computational modeling as a flexible and valuable approach to generate insights and complement medical practice with a strong mechanistic perspective. However, in many cases, the effective translation of these models into clinical settings and their widespread adoption in medical practice require further personalization to account for inter-individual variability (Zhao et al. 2002; Salat et al. 2012; Schutte et al. 2022). This is particularly challenging in the domain of 1D blood flow modeling, as these models require the definition of several parameters, and acquiring data to support the calibration process of these models is difficult. Moreover, when acquired, data involves time-dependent signals which can be noisy and highly variable at the beat-to-beat scale.

From a mathematical perspective, the transition from generic to subject-specific blood flow models typically involves solving an inverse problem, in which model input parameters are adjusted to ensure that the model best reproduces patient data. A typical example is the calibration of Windkessel boundary conditions using physiological measurements such as cardiac output, invasive fractional flow reserve, or blood flow distribution among vascular regions. This problem has been successfully addressed through variations of quasi-Newton algorithms (Olufsen et al. 2002; Grinberg and Karniadakis 2008; Spilker and Taylor 2010; Troianowski et al. 2011; Blanco et al. 2012) and approaches based on Gaussian processes (Perdikaris and Karniadakis 2016; Yin et al. 2019; Borowska et al. 2022). Methods derived from the Kalman filter have also been proposed for the estimation of boundary conditions (DeVault et al. 2008; Lal et al. 2017), mechanical properties (Bertoglio et al. 2012; Pant et al. 2014; Lombardi 2014), and the incorporation of measured waveforms in both time and frequency domains (Moireau and Chapelle 2011; Caiazzo et al. 2017; Müller et al. 2019). More recently, algorithms based on evolutionary strategies have emerged as powerful tools for high-dimensional optimization problems. For instance, in (Dumas et al. 2017), the authors constructed a patient-specific model of the lower limb circulation using non-invasive measurements of lumen area and velocity profiles acquired in nearly every vessel of the network. Similarly, in (Molléro et al. 2018), a multifidelity cardiac model was optimized to reproduce subject-specific features such as stroke volume and mean and diastolic aortic pressures, by applying an evolutionary strategy to estimate scalar parameters that included contractility and stiffness.

In this work, we address the problem of calibration of 1D models of the systemic circulation to match patient-specific pressure and flow rate measurements. To achieve this, two main objectives are defined. The first goal is to perform an exhaustive global sensitivity analysis (over the mechanical, geometrical and boundary condition parameters) to quantify the impact of local variations of each model parameter on the predicted waveforms, therefore identifying the ones of utmost importance in the calibration process. The second goal is to propose a parameter identification approach to calibrate the 1D model to match the patient-specific measurements. In this regard, an evolutionary strategy named as CMA-ES (Hansen et al. 2003) is employed to estimate the cardiac ejection waveform and the sets of parameters identified as having high, mid and low importance according to the previous sensitivity analysis. The optimisation process is carried out through a simultaneous multi-parameter (all-at-once) approach, allowing us to explore complex parameter combinations and therefore capture nonlinear interactions among mechanical parameters while accounting for their regional variability across the vascular network. Despite the challenges posed by the large-scale, high-dimensional, and small-data nature of the problem, the optimized model predictions satisfactorily match subject-specific measurements while maintaining physiologically consistent behavior along different vascular territories.

The document is organized as follows. Section 2 describes in detail the components and parameters of the 1D cardiovascular model. The terminal, geometric, and mechanical parameters are presented, along with the physiological ranges in which they lie. This section also introduces the available patient data and the similarity metrics used to guide the optimization process. Section 3 is devoted to the detection of unimportant parameters through global sensitivity analysis, intending to reduce the dimensionality of the identification problem. In Sect. 4, the Covariance Matrix Adaptation Evolutionary Strategy (CMA-ES) is used for model personalization. The optimization is performed considering both the complete set of parameters and the subset identified as most relevant by the sensitivity analysis, thereby verifying the reduction approach proposed in the previous section. Additionally, the stochastic nature of the evolutionary strategy and its impact on the optimization process are examined. Section 5 summarizes the main findings of this work, emphasizing their implications and limitations, while Sect. 6 outlines the final remarks.

## Materials and methods

This section describes the 1D blood flow network model used as a model substrate in the identification process. We employ a simplified version of the Anatomically Detailed Arterial Network (ADAN) model (Blanco et al. 2015), called ADAN-86, including the 86 most representative arterial segments of the arterial circulation (Blanco et al. 2020; Boileau et al. 2015). We present the governing equations and the model parameters that characterize geometric and mechanical features, as well as the inlet/outlet boundary conditions in the problem. For further details, see (Watanabe 2013; Blanco et al. 2014).

### The ADAN-86 model

For a single vascular segment, the set of equations corresponding to the 1D blood flow theory is:

1 $$\begin{aligned} \dfrac{\partial A}{\partial t} + \dfrac{\partial q}{\partial x} = 0, \qquad \dfrac{\partial q}{\partial t} + \dfrac{\partial }{\partial x}\left( \dfrac{q^2}{A}\right) + \dfrac{A}{\rho } \dfrac{\partial p}{\partial x} = -\dfrac{8\pi \mu }{\rho }\dfrac{q}{A}, \end{aligned}$$

where *A*(*x*, *t*) is the cross-sectional area of the vessel, *q*(*x*, *t*) the mass flow rate, *p*(*x*, *t*) the average blood pressure in the cross section, $\mu$ and $\rho$ the dynamic viscosity and density of blood, respectively. This set of equations is defined in the space-time domain $[0,L]\times [0,T]$, with *L* the vessel length and *T* the final temporal instant. The system is closed by introducing a constitutive equation relating the pressure to the vessel deformation (see Equation (5) below).

**Fig. 1:**
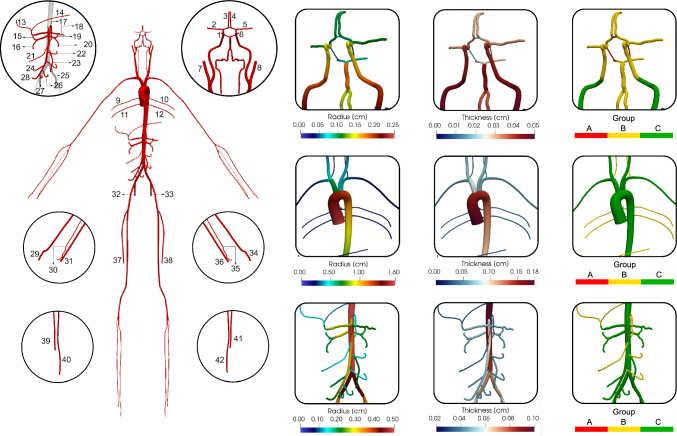
Description of arterial geometry, with details in regions of the head, aortic arch, and abdomen of the ADAN-86 model. Color-coded panels feature the vessel radius, arterial wall thickness, and division into regional groups according to the vessel radius (see Sect. 2.2.3)

In the ADAN-86 model, the 1D blood flow equations are applied within an anatomically realistic topological representation of the entire arterial tree. As boundary conditions, the model requires the definition of the flow rate at the aortic root, while terminal vessels are coupled to lumped-parameter models representing the peripheral circulation. At network junctions, the conservation of mass and total pressure are considered as follows:

2 $$\begin{aligned} \sum _{i = 1}^{N_T} Q_i = 0, \qquad p_1 + \dfrac{\rho }{2} \left( \dfrac{q_1}{A_1}\right) ^2 = p_i + \dfrac{\rho }{2} \left( \dfrac{q_i}{A_i}\right) ^2, \, i = 1, \ldots , N_T, \end{aligned}$$

being $N_T$ the number of converging arterial segments. A geometric representation of the model, with emphasis on the head, aortic arch, and abdominal regions, is shown in Fig. 1. In this figure, the 42 terminal segments are enumerated, and details of arterial radius and wall thickness are provided through color-coded panels. The rightmost column features a regional division of the network into groups, which will be employed to characterize the mechanical behavior of each arterial segment. A detailed description of each parameter in the ADAN-86 model, the applied allometric relations, and their values for an average subject are reported in (Blanco et al. 2015, 2020). To keep this document as self-contained as possible, we briefly describe the model parameters below, grouped according to their nature into geometric, terminal, material, and inflow parameters.

### Model parameters

#### Geometric parameters

The length (*L*) and the reference radius ($r_0$) of each arterial segment in the ADAN-86 model were determined after an exhaustive review of the anatomical, clinical, and surgical literature. This process, detailed in (Watanabe 2013), defines the morphology of an average subject according to state-of-the-art literature. For the wall thickness (*h*), based on the measurements reported in (Avolio 1980), a curve fitting procedure was applied to obtain the following relation:

3 $$\begin{aligned} h = r_0 \left( a\exp ^{br_0} + c\exp ^{dr_0} \right) , \end{aligned}$$

with $a = 0.2802$, $b = -5.053\,$cm$^{-1}$, $c = 0.1324$ and $d = -0.1114\,$cm$^{-1}$. This expression is widely used in the literature, as can be seen in (Boileau et al. 2015; Heltai et al. 2021; Padmos et al. 2022). Using this relation, the wall thickness ranges from 0.01 cm, in the more distal segments of the head circulation, to 0.18 cm in the aortic arch, as can be seen in Fig. 1.

#### Terminal parameters

To represent the effect of the peripheral beds in terms of boundary conditions to the 1D model, each terminal vessel is coupled to a three-element Windkessel model, where the relation between pressure and flow rate reads:

4 $$\begin{aligned} R_A R_B C \dfrac{d q}{dt} = R_B C\dfrac{d}{dt}(p - p_T) + ( p - p_T) - (R_A + R_B)q, \end{aligned}$$

where $p_T$ is a reference terminal pressure, assumed to be zero, *C* is the peripheral compliance, and $R_A$ and $R_B$ are resistive elements for arterioles and capillaries vessels, respectively. For the ADAN-86 model, the parameters for each Windkessel element are reduced to compliance (*C*) and a single resistance (*R*), defining the total resistance $R = R_A + R_B$ and assuming $R_A = 0.2R$. These parameters are computed such that the blood supply to the peripheral bed is in agreement with the literature and with a physiological pressure range (Blanco et al. 2012, 2014). Terminal elements are enumerated in Fig. 1 and the resistance and compliance values are detailed in Table 1.

**Table 1:** Baseline values for the Windkessel parameters for each terminal vessel in the ADAN-86 model

Region	No	Name	Reference	Resistance ($\times 10^{4}$)		Compliance
			radius	$R_A$	$R_B$	($\times 10^{-6}$)
Head	1	R. Posterior Communicating	0.0869	2.6297	10.5188	1.2611
	2	R. Middle Cerebral	0.1041	1.0714	4.2856	3.4020
	3	R. Anterior Cerebral	0.0965	1.2196	4.8784	2.7941
	4	L. Anterior Cerebral	0.0965	1.2196	4.8784	2.7941
	5	L. Middle Cerebral	0.1041	1.0714	4.2856	3.4020
	6	L. Posterior Communicating	0.0869	2.6297	10.5188	1.2611
	7	L. External Carotid	0.2265	0.8541	3.4167	6.0105
	8	R. External Carotid	0.2265	0.8511	3.4047	6.0317
Thorax	9	R. Intercostal T6	0.1400	22.5808	90.3234	0.2273
	10	L. Intercostal T6	0.1400	23.1660	92.6642	0.2216
	11	R. Intercostal T7	0.1550	21.0677	84.2711	0.2436
	12	L. Intercostal T7	0.1550	21.2483	84.9933	0.2416
Abdomen	13	R. Hepatic artery	0.1420	0.4714	1.8857	10.8900
	14	L. Hepatic artery	0.1166	0.8520	3.4083	6.0253
	15	R. Superior segmental	0.1927	0.5278	2.1112	9.7268
	16	R. Inferior segmental	0.1927	0.5278	2.1112	9.7268
	17	L. gastric	0.1506	31.1250	124.5002	0.1649
	18	Splenic artery	0.2166	0.4290	1.7161	10.1967
	19	L. Superior segmental	0.1927	0.5260	2.1042	9.7595
	20	L. Inferior segmental	0.1927	0.5260	2.1042	9.7595
	21	Middle colic	0.1425	2.6865	1.0746	1.9111
	22	Jejunal T10	0.1580	1.9695	7.8780	2.6067
	23	Inferior mesentric T5	0.2077	2.1674	8.6698	2.3687
	24	Ileocolic T9	0.2000	0.9717	3.8868	5.2835
	25	Jejunal T11	0.1580	1.9695	7.8780	2.6067
	26	Ileal T12	0.1801	1.3296	5.3185	3.8612
	27	Ileal T13	0.1801	1.3296	5.3185	3.8612
	28	Superior mesentric T4	0.2067	0.8807	3.5229	5.8292
Upper Limbs	29	R. Radial	0.1378	1.0458	4.1835	4.9088
	30	R. Posterior interosseuous	0.0676	4.3337	17.3351	1.1847
	31	R. Ulnar	0.1408	1.0648	4.2595	4.8212
	34	L. Radial	0.1378	1.0271	4.1085	4.9983
	35	L. Posterior interosseuous	0.0676	4.3494	17.3979	1.1804
	36	L. Ulnar	0.1408	1.0855	4.3421	4.7295
Lower Limbs	32	R. Internal Iliac	0.2818	0.3757	1.5030	10.3663
	33	L. Internal Iliac	0.2818	0.3768	1.5075	10.3623
	37	R. Profunda Femoris	0.2144	0.3106	1.2424	10.6529
	38	L. Profunda Femoris	0.2144	0.3107	1.2431	10.6529
	39	R. Anterior Tibial	0.1166	2.2229	8.8918	2.3096
	40	R. Posterior Tibial	0.1229	1.9176	7.6704	2.6773
	41	L. Anterior Tibial	0.1166	2.2236	8.8946	2.3088
	42	L. Posterior Tibial	0.1229	1.9184	7.6737	2.6761

Terminal numbering corresponds to Fig. 1. Reference vessel radius (in cm), terminal resistances (in dyn s $\textrm{cm}^{-5}$), and terminal compliances (in $\textrm{cm}^5$ $\textrm{dyn}^{-1}$) are reported

#### Constitutive parameters

To relate the pressure to the vessel wall deformation, the following constitutive law is considered:

5 $$\begin{aligned} & p(x,t) = p_0 + \dfrac{\pi h r_0}{A} \Bigl [ E_e \varepsilon + \varepsilon _r E_c \ln ( e^u + 1 ) + K_m\dot{\varepsilon } \Bigr ], \nonumber \\ & \qquad u = \dfrac{\varepsilon - \varepsilon _0}{\varepsilon _r}, \qquad \varepsilon =\sqrt{\frac{A}{A_0}}-1, \end{aligned}$$

with $p_0$ accounting for a homeostatic equilibrium pressure, $r_0 = r_0(x)$, $A_0 = A_0(x)$ and $h_0 = h_0(x)$ are the vessel radius, its associated cross-sectional area and vessel wall thickness at the reference state, respectively, and $\varepsilon = \varepsilon (A,A_0)$ is the current vessel deformation. This relation, inspired by (Armentano et al. 1991), describes the parallel arrangement of elastin, collagen, and smooth muscle. In the same relation, $E_e = E_e(x)$ and $E_c = E_c(x)$ are the effective Young modulus of the elastin and collagen, respectively, and $K_m$ is the effective viscoelastic parameter. Finally, following the ideas proposed in (Urquiza et al. 1995), $\varepsilon _0 = \varepsilon _0(A_0)$ is the deformation state for which $50\%$ of collagen fibers have been activated and $\varepsilon _r = \varepsilon _r(A_0)$ is the standard deviation of the fiber activation state distribution. For each arterial vessel, the material parameters ($E_e, E_c, K_m, \varepsilon _0, \varepsilon _r$) are defined according to the vessel size. This classification is intended to clearly distinguish between small, predominantly muscular arteries (Group A), medium-sized arteries containing both elastin and collagen (Group B), and predominantly elastic large arteries (Group C). These groups are depicted in the rightmost panel of Fig. 1, while the material property values for each group are provided in Table 2.

**Table 2:** Baseline values for the material parameters based on regional groups

Group	Radius	$E_e$ [dyn/cm$^2$]	$E_c$ [dyn/cm$^2$]	$K_m$ [dyn/cm$^2$]	$\varepsilon _0$	$\varepsilon _r$
[A] Small arteries	$< 0.07$	$1.8\times 10^{6}$	0	$1.65\times 10^{5}$	0.35	0.05
[B] Mid-size arteries	[0.07, 0.18]	$2.6\times 10^{6}$	$0.20\times 10^{9}$	$0.45\times 10^{5}$	0.35	0.05
[C] Large arteries	$> 0.18$	$3.4\times 10^{6}$	$0.05\times 10^{9}$	$0.30\times 10^{5}$	0.35	0.05

#### Inflow parameters

**Fig. 2:**
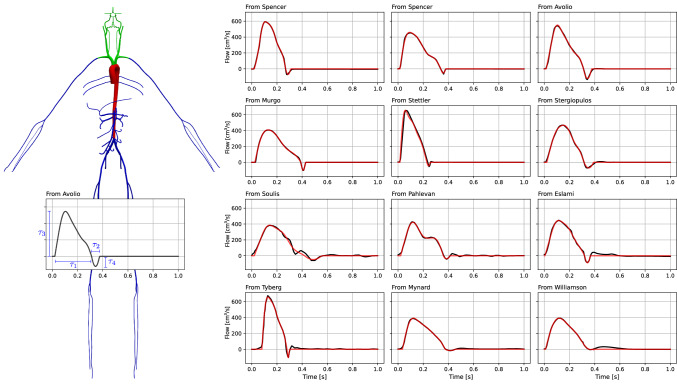
Comparison of the waveform morphing protocol to define the inflow boundary condition to the ADAN-86 model and eleven measured signals of the cardiac ejection waveform. In each panel, the red curve stands for the proposed morphing flow waveform strategy, and in black the waveform reported in the corresponding reference. On the left panel, arterial vessels are colored according to the division for the regionalized parameter identification: aorta segments (red), head and neck segments (green), and remaining vascular segments (blue)

At the inlet of the arterial system, at the aortic root, a boundary condition that prescribes the incoming flow rate into the ADAN-86 model is considered. To allow for inter-individual variability, it is essential to define a strategy to parametrize this inflow boundary condition, which is defined by a time-series flow signature defined by 200 data points (time vs. flow rate). For the baseline ADAN-86 model, this waveform was taken from (Murgo et al. 1980) and scaled to achieve a cardiac output of $Q = 6.72$ L/min and a heart rate of 60 bpm. In the present work, a ten-parameter morphing strategy is proposed such that the flow waveform can be modified, creating different waveforms. The waveform scaling is controlled by four parameters: $\tau _1$ and $\tau _2$ specify the duration (in seconds) of the ejection and regurgitation phases, respectively, while $\tau _3$ and $\tau _4$ define the corresponding peak values (in cm$^3$/s), as can be seen in the left part of Fig. 2. The shape of the ejection phase is obtained from the inverse of the discrete sine transform (DST) of a frequency vector $\omega \in {\mathbb {R}}^{200}$, where only the first six components are considered nonzero. The shape of the regurgitation phase is assumed parabolic, fully determined by $\tau _2$ and $\tau _4$. After the regurgitation phase, zero flow is imposed. This simple parametrization strategy has proven effective for describing a variety of ventricular ejection waveforms. As an illustration of its versatility, Fig. 2 depicts the corresponding parametric approximations considering as targets the measured waveforms labeled as Spencer (Spencer and Denison 1959), Avolio (Avolio 1980), Murgo (Murgo et al. 1980), Stettler (Stettler et al. 1981), Stergiopulos (Stergiopulos et al. 1992), Soulis (Soulis et al. 2011), Pahlevan (Pahlevan and Gharib 2014), Eslami (Eslami et al. 2019), Tyberg (Tyberg 2021), Mynard (Mynard et al. 2023), and Williamson (Williamson et al. 2024).

This parametrization strategy extends the approach proposed in Müller et al. (2019), in which only the first four parameters were considered, by allowing changes in waveform morphology in addition to the amplitude and duration of the antegrade and retrograde phases. Despite the higher number of parameters, this more detailed parametrization is necessary to correctly represent the impact of a large variability of cardiovascular alterations, such as cardiac or valvular dysfunction, on aortic flow wave morphology.

#### Parameter setting

The setting of model parameters is performed as described in the previous section, and this setting is regarded as the baseline configuration. Along with the parameter identification procedure, each model parameter is varied from its baseline value by introducing a multiplicative factor, as detailed in Table 3. For the geometric parameters, the range of each scale factor is defined to encompass the deviations from baseline values reported in the literature. The deviation of the baseline radius relative to patient-specific geometries is reported in (Watanabe 2013), while vessel length variation ranges to represent subjects whose total heights vary between 161.5 cm and 190.4 cm (Chen et al. 2016). For the terminal parameters, a heuristic definition of the variation range is adopted, assuming an ideal linear relationship between total resistance and flow distribution. Finally, for the parameters related to the constitutive arterial wall equation (5), the ranges are defined according to the deviations from baseline reported in (Avolio 1980; Armentano et al. 1991; Blanco et al. 2015).

In summary, the 1D blood flow model is characterized by the parameters listed in Table 3. In this table, the baseline values correspond to those used in the reference ADAN-86 model. For the cardiac ejection morphing strategy, the baseline values correspond to the ADAN-86 reference boundary condition, and the range of each multiplicative factor is defined based on a simple parameter exploration to ensure that the waveforms reported in Fig. 2 were accurately reproduced. It is worth noting that the first three parameter types (i.e., geometric, terminal, and constitutive) can be adjusted either at the vessel level, by assigning a distinct scale factor to each artery, or at a more global level, by applying the same scaling factor to groups of vascular vessels. In the present work, we consider a grouped modification of these model parameters for the groups of vessels displayed in the ADAN-86 illustration seen in Fig. 2. These three groups correspond to the aorta (red segments in the figure), the vessels in the neck and head (green segments in the figure), and the remaining vessels (blue segments in the figure). This methodological aspect is described in more detail in the next section.

**Table 3:** Modifiable parameters in the ADAN-86 model

Type	Model parameter				Multiplicative factor	
	Name	Symbol	Unit	Base value	Symbol	Range
Geometry	Vessel length	*L*	cm		$\theta _L$	[0.95, 1.12]
	Lumen radius	$r_0$	cm		$\theta _r$	[0.80, 1.40]
	Arterial wall thickness	*h*	cm	Equation (3)	$\theta _h$	[0.80, 1.40]
Terminal	Total resistance	*R*	dyn s $\textrm{cm}^{-5}$	Table 1	$\theta _R$	[0.25, 2.00]
	Compliance	*C*	$\textrm{cm}^5$ $\textrm{dyn}^{-1}$	Table 1	$\theta _C$	[0.80, 2.50]
Constitutive	Elastin	$E_e$	dyn $\textrm{cm}^{-2}$	Table 2	$\theta _{Ee}$	[0.85, 4.00]
	Collagen	$E_c$	dyn $\textrm{cm}^{-2}$	Table 2	$\theta _{Ec}$	[0.80, 1.50]
	Viscoelasticity	$K_m$	dyn $\textrm{cm}^{-2}$	Table 2	$\theta _{K}$	[0.10, 1.20]
	Collagen activation mean	$\varepsilon _0$		0.35	$\theta _{\varepsilon 0}$	[0.50, 1.15]
	Collagen activation width	$\varepsilon _r$		0.05	$\theta _{\varepsilon r}$	[0.80, 2.00]
Cardiac ejection	Antegrade duration	$\tau _1$	s	0.3657	$\theta _{\tau 1}$	[0.50, 1.20]
	Retrograde duration	$\tau _2$	s	0.0352	$\theta _{\tau 2}$	[0.28, 8.00]
	Antegrade amplitude	$\tau _3$	$\textrm{cm}^3$ $\textrm{s}^{-1}$	546.88	$\theta _{\tau 3}$	[0.60, 1.50]
	Retrograde amplitude	$\tau _4$	$\textrm{cm}^3$ $\textrm{s}^{-1}$	$-136.01$	$\theta _{\tau 4}$	[0.00, 1.20]
	First DST amplitude	$\omega _1$	$\textrm{cm}^3$ $\textrm{s}^{-1}$	961.65	$\theta _{\omega 1}$	[0.50, 1.20]
	Second DST amplitude	$\omega _2$	$\textrm{cm}^3$ $\textrm{s}^{-1}$	362.10	$\theta _{\omega 2}$	[0.25, 1.20]
	Third DST amplitude	$\omega _3$	$\textrm{cm}^3$ $\textrm{s}^{-1}$	138.08	$\theta _{\omega 3}$	$[-0.2, 1.40]$
	Fourth DST amplitude	$\omega _4$	$\textrm{cm}^3$ $\textrm{s}^{-1}$	13.952	$\theta _{\omega 4}$	$[-3.5, 7.00]$
	Fifth DST amplitude	$\omega _5$	$\textrm{cm}^3$ $\textrm{s}^{-1}$	36.998	$\theta _{\omega 5}$	$[-0.5, 2.00]$
	Sixth DST amplitude	$\omega _6$	$\textrm{cm}^3$ $\textrm{s}^{-1}$	$-5.220$	$\theta _{\omega 6}$	$[-8.0, 5.00]$

Baseline values for the cardiac ejection parametrization correspond to the reference ADAN-86 ejection waveform

### Data acquisition

The study population consists of 11 patients from the Hospital Israelita Albert Einstein (São Paulo, Brazil) with suspected or diagnosed coronary artery disease. For each patient, the collected data includes velocity waveform in the common carotid artery and pressure signals in the aortic arch, the radial and subclavian arteries. Pressure waveforms were recorded during coronary angiography using TEB hemodynamic polygraph system (TEB, São Paulo, Brazil) equipped with the SPPOS software, version 9.0, revision 6. All invasive signals were continuously acquired at a sampling frequency of 1 kHz and digitally stored for subsequent analysis. Ultrasound images were obtained using a LOGIQ E(R7) ultrasound system (GE Healthcare, Chicago, IL, USA). Flow velocity profiles and lumen area measurements were acquired from the common carotid artery using a high-frequency linear probe, and were converted into the final flow rate waveform used in subsequent analysis. All measurements were performed by experienced operators under standardized acquisition conditions and anonymized before numerical processing. All the procedures were approved by the Ethical Committee.

The study included data from 7 men and 4 women, with a mean age of $60 \pm 8$ years (range $45-77$). The mean body weight was $76 \pm 19$ kg, and the average height was $164 \pm 10$ cm. Regarding cardiovascular risk factors, arterial hypertension was present in $90\%$ of the patients, diabetes mellitus in $72\%$, and dyslipidemia in $45\%$. The extent of coronary artery disease (number of affected vessels) was distributed as follows: one-vessel in $36\%$, two-vessel in $27\%$, and three-vessel disease in $27\%$ of patients. The mean left ventricular ejection fraction (LVEF) was $54.87 \pm 7.66\%$, and the SYNTAX score, which quantifies the complexity and severity of coronary artery disease based on angiographic findings Neumann et al. (2019), ranged from 0 to 29. A detailed description on a per patient basis is listed in Table 4.

**Table 4:** Demographic information for the study cohort

ID	Sex	Age	Weight	Height	LVEF	DAC	SYNTAX	Hyp	Diab	Dys
		[years]	[kg]	[cm]	[$\%$]		index			
01	M	56	120	177	-	2	7	0	0	0
02	F	45	58	156	64	1	10	1	1	1
03	M	63	65	168	47	1	2	1	1	1
04	M	60	77	168	62	3	29	1	1	0
05	M	57	68	153	59	3	15	1	1	0
06	M	56	86	178	45	1	7	1	0	0
07	F	66	66	155	47	2	16	1	1	1
08	F	53	75	160	-	3	25	1	1	1
09	M	77	52	176	61	1	16	1	0	0
10	M	56	95	168	54	2	27	1	1	0
11	F	69	75	150	-	0	0	1	1	1
Mean ± std		$60 \pm 8$	$76\pm 19$	$164 \pm 10$	$54.9 \pm 7.7$					
Class: Prevalence						$1:36\%$	$\le 10:36\%$	$1:90\%$	$1:72\%$	$1:45\%$
						$2:27\%$	$>20:27\%$	$0:10\%$	$0:18\%$	$0:55\%$
						$$3:27\%$$				

For each patient, identified by their patient ID, information on sex, age (in years), weight (in kg), height (in cm), mean left ventricular ejection fraction (LVEF), the number of affected vessels (Diseased Artery Count - DAC) and SYNTAX index, is provided. Furthermore, the presence (coded by 1) or absence (coded by 0) of hypertension (Hyp), diabetes mellitus (Diab) and dyslipidemia (Dys) is reported

**Fig. 3:**
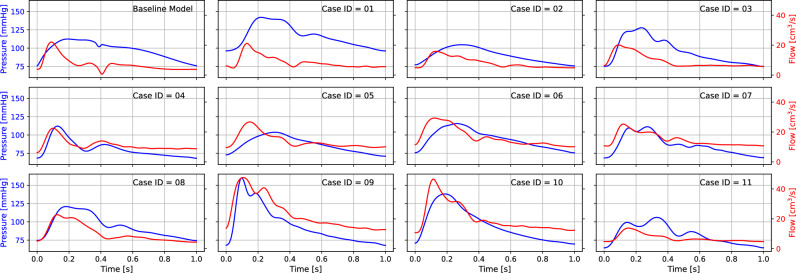
Comparison of flow rate and pressure waveforms as given by the predictions of the baseline ADAN-86 model, and each patient-specific signal from the cohort of eleven patients. In each panel, the red curve stands for the flow rate waveform in the common carotid artery, while the blue curve stands for the pressure waveform in the aortic arch

In this work, to avoid potential bias in interpreting the results due to unbalanced information, we consider only the carotid flow and aortic arch pressure signals for each patient. These signals, hereafter identified by an ID from 1 to 11, together with the baseline model solution, are shown in Fig. 3. It is important to note that the measured waveforms have not been post-processed to remove potential noise or account for calibration issues in the clinical instruments. Patient data exhibit substantial deviations from the baseline model in both waveform shape and amplitude, underscoring the challenges of the optimization task.

### Optimization setup

To capture local intra-individual variability within the arterial system, the vasculature is divided into three regions, each characterized by distinct mechanical properties and defined according to available data: (i) aortic vascular segments, which predominantly determine global compliance and serve as the location of the measured pressure waveform; (ii) head and neck vascular segments, extending from the neck vessels to the intracranial circulation, where the flow rate waveform was recorded; and (iii) the remaining arteries of the limbs and abdominal organs. This three-group subdivision, illustrated in Fig. 2, is responsible for targeting specific regional-based parameter modifications. That is, the parameters corresponding to all the vessels pertaining to a certain group are equally affected through the optimization procedure. For each region (aorta, head, and limbs/organs), 10 parameters are defined to represent multiplicative factors for the geometric, terminal, and constitutive properties (see Table 3). In addition, we have the 10 parameters used to characterize the cardiac ejection waveform. So, overall, the problem involves 40 model parameters. It is important to note that all terminal vessels are assigned either to the head or limbs/organ regions; thus, terminal parameters (resistance and compliance) associated with the aorta region play no role in the calibration procedure, and do not affect model predictions. For the sake of consistency, these two parameters are retained in our analysis. Hereafter, we will denote the complete set of scale factor parameters by $\boldsymbol{\theta } \in {\mathbb {R}}^n$.

To quantify the discrepancy between the model predictions and the patient measurements, we adopt the conventional Euclidean norm of the difference between time-discretized signals. Let ${\mathcal {Q}}$, ${\mathcal {P}} \in {\mathbb {R}}^N$ denote the measured carotid flow rate and aortic pressure waveforms, respectively, and ${\mathcal {Q}}^\text {m}(\boldsymbol{\theta })$, ${\mathcal {P}}^\text {m}(\boldsymbol{\theta })\in {\mathbb {R}}^N$ their model-predicted counterparts for a given scale factor parameter $\boldsymbol{\theta } \in {\mathbb {R}}^n$. The mismatch, or cost function, is then defined as:

6 $$\begin{aligned} {\mathcal {F}}(\boldsymbol{\theta }) = \left[ \dfrac{1}{\overline{{\mathcal {Q}}}^2} \left\| {\mathcal {Q}} - {\mathcal {Q}}^\text {m}(\boldsymbol{\theta }) \right\| _N^2 + \dfrac{1}{\overline{{\mathcal {P}}}^2} \left\| {\mathcal {P}} - {\mathcal {P}}^\text {m}(\boldsymbol{\theta }) \right\| _N^2 \right] ^{1/2}, \end{aligned}$$

with $\left\| \cdot \right\| _N$ the usual $\ell ^2$-norm in ${\mathbb {R}}^N$ and $\overline{(\cdot )}$ the corresponding mean value. The mismatch between the baseline ADAN-86 predicted waveforms, and each patient measured waveforms, is reported in Fig. 3. These values were computed for a uniform time-discretization with $N = 200$.

## Global sensitivity analysis

### Sobol sensitivity indices

Within the context of model calibration procedures, it is important to identify which parameters exert the greatest influence on the variability of the mismatch between model predictions and measurements, that is, the sensitivity of the cost function to model parameters. As described in (Saltelli et al. 2004), sensitivity analysis (SA) examines how uncertainty in a model’s output (numerical or otherwise) can be attributed to different sources of uncertainty in model inputs. A widely used approach to perform SA is the computation of Sobol indices, a variance decomposition method that quantifies the proportion of output variance attributable to each input or combination of inputs (Soboĺ 1993; Sobol 2001). This technique, popular for complex and nonlinear models, has recently been applied in various computational hemodynamics studies (Chen et al. 2013; Campos et al. 2020; Xu et al. 2021; Sala et al. 2023).

In a few words, let the input parameters $\boldsymbol{\theta } = \{\theta _i\}\in {\mathbb {R}}^n$ be random independent variables following each a probability distribution, employed to compute the random output (cost function) ${\mathcal {F}}$. The first (*F*) and the total (*T*) Sobol indices are, respectively, defined as:

7 $$\begin{aligned} F_i = \dfrac{\text {var}\left[ {\mathbb {E}}({\mathcal {F}}|\theta _i) \right] }{\text {var}\left[ {\mathcal {F}} \right] } \qquad T_i = 1 - \dfrac{\text {var}\left[ {\mathbb {E}}({\mathcal {F}}|\theta _{-i}) \right] }{\text {var}\left[ {\mathcal {F}} \right] }, \qquad i=1,\ldots ,n. \end{aligned}$$

Here, as usual, $\text {var}\left[ \cdot \right]$ denotes the variance, ${\mathbb {E}}$ the expected value and $\theta _{-i} = \left( \theta _1, \cdots , \theta _{i-1}, \theta _{i+1}, \cdots , \theta _{n}\right)$ aims to take over all possible values of $\boldsymbol{\theta }$ while keeping $\theta _i$ fixed. While the first-order Sobol indices represent the portion of the variance of the output caused by the *i*-th uncertain input, the total index introduces possible effects due to higher-order interactions of the *i*-th and the remaining inputs. When $T_i \approx 0$, it means that $\theta _i$ has low influence on the output and could be fixed in its range of uncertainty.

At discrete level, first and total Sobol indices are efficiently computed through their Monte Carlo estimators. Let $N_s$ be an integer value representing the sampling space. Two matrices of size $N_s \times n$ are defined as:

8 $$\begin{aligned} {\textbf{A}} = \begin{bmatrix} \theta _{1}^{(1)} & \theta _{2}^{(1)} & \ldots & \theta _{n}^{(1)} \\ \theta _{1}^{(2)} & \theta _{2}^{(2)} & \ldots & \theta _{n}^{(2)} \\ \vdots & \vdots & & \vdots \\ \theta _{1}^{(N_s)} & \theta _{2}^{(N_s)} & \ldots & \theta _{n}^{(N_s)} \\ \end{bmatrix}, \qquad {\textbf{B}} = \begin{bmatrix} \eta _{1}^{(1)} & \eta _{2}^{(1)} & \ldots & \eta _{n}^{(1)} \\ \eta _{1}^{(2)} & \eta _{2}^{(2)} & \ldots & \eta _{n}^{(2)} \\ \vdots & \vdots & & \vdots \\ \eta _{1}^{(N_s)} & \eta _{2}^{(N_s)} & \ldots & \eta _{n}^{(N_s)} \\ \end{bmatrix}, \end{aligned}$$

where $\eta _i^{(k)}$ denotes an independent sample of parameters $\theta _i$, draw from the same probability distribution but independently from $\theta _i^{(k)}$. For each $i = 1, \ldots , n$, a matrix ${\textbf{A}}_{{\textbf{B}}}^{(i)}$ is assembled by replacing the $i-$th column of ${\textbf{A}}$ with the corresponding column of ${\textbf{B}}$, as

9 $$\begin{aligned} {\textbf{A}}_{{\textbf{B}}}^{(i)} = \begin{bmatrix} \theta _{1}^{(1)} & \theta _{2}^{(1)} & \ldots & \eta _{i}^{(1)} & \ldots & \theta _{n}^{(1)} \\ \theta _{1}^{(2)} & \theta _{2}^{(2)} & \ldots & \eta _{i}^{(2)} & \ldots & \theta _{n}^{(2)} \\ \vdots & \vdots & & \vdots \\ \theta _{1}^{(N_s)} & \theta _{2}^{(N_s)} & \ldots & \eta _{i}^{(N_s)} & \ldots & \theta _{n}^{(N_s)} \\ \end{bmatrix}. \end{aligned}$$

Note that the total number of model evaluations required is $N = (n + 2)N_s$, $N_s$ evaluations for each ${\textbf{A}}$ and ${\textbf{B}}$, and additional $nN_s$ evaluations for ${\textbf{A}}_{{\textbf{B}}}^{(i)}$. On top of this hybrid matrix, the Sobol indices are approximated as

10 $$\begin{aligned} & F_i \approx \dfrac{1}{\text {varF}}\dfrac{1}{N_s}\sum _{k = 1}^{N_s} Y_B^{(k)}\left( Y^{(k)}_{A_B^i} - Y_A^{(k)} \right) , \nonumber \\ & \qquad T_i \approx \dfrac{1}{\text {varF}}\dfrac{1}{2N_s} \sum _{k = 1}^{N_s} \left( Y_A^{(k)} - Y^{(k)}_{A_B^i} \right) ^2, \end{aligned}$$

where $Y_A^{(k)} = {\mathcal {F}}({\textbf{A}}_k)$, $Y_B^{(k)} = {\mathcal {F}}({\textbf{B}}_k)$, $Y^{(k)}_{A_B^i} = {\mathcal {F}}(({\textbf{A}}_{{\textbf{B}}}^{(i)})_k)$, and varF approximates the total variance as:

11 $$\begin{aligned} \text {var}[{\mathcal {F}}] \approx \text {varF} = \dfrac{1}{N_s}\sum _{k = 1}^{N_s} \left( Y_A^{(k)} \right) ^2 - \left( \dfrac{1}{N_s} \sum _{k = 1}^{N_s} Y_A^{(k)}\right) ^2. \end{aligned}$$

In (Xu et al. 2021), the authors state that there is a common consensus in the computational hemodynamics community that geometry and boundary conditions play a more significant role in blood flow modelling than rheological behavior or fluid–structure interaction. In this context, we focus on ranking the influence of each model parameter on the cost function, excluding those that define the boundary conditions, that is, the parameters affecting the cardiac ejection waveform and the terminal resistances. To minimize bias in the parameter ranking caused by enforcing a specific cardiac ejection waveform, the SA is repeated using two additional cardiac ejection boundary conditions. Furthermore, this process is performed for each patient measurement to analyze inter-individual variability.

### Parameter sensitivity

The SA is performed employing the SALib library (Herman and Usher 2017; Iwanaga et al. 2022). By keeping fixed the parameters related to the cardiac ejection waveform ($\theta _\tau$ and $\theta _\omega$) and the terminal resistances ($\theta _R$), the number of parameters is equal to $n = 27$. All the parameters were assumed to follow independent uniform distributions within the ranges specified in Table 3, reflecting the absense of prior information favoring specific parameter values. The sample space is discretized from the Saltelli sequence (Saltelli 2002; Saltelli et al. 2004) with size $N_s = 1\,200$, which results in a total of 35K samples. The convergence of first-order and total sensitivity indices was verified to ensure variations less than $1\%$.

The computational cost, for first and total order Sobol indices, is $(n + 2)N_s$ model evaluations, where $n = 27$ is the dimension of the parameter space and $N_s$ the sampling size. From a computational point of view, each sample evaluation was run with a time step of $\Delta t = 0.001$ seconds, heart rate of 60 bpm, for a total of 10 cardiac cycles to ensure the convergence to the periodic state. Sampling evaluations were carried out on the Santos Dumont supercomputer facility, exploiting the highly parallel nature of the SA procedure, using a single node (AMD Genoa-X 9684X) with 192 MPI processes, with an average run time per sample of 860 seconds, and a total run time of 45 hours for each cardiac waveform case. The resulting dataset, with 35K samples, is employed to quantify the sensitivity of the cost function associated with each patient to the model parameters.

**Fig. 4:**
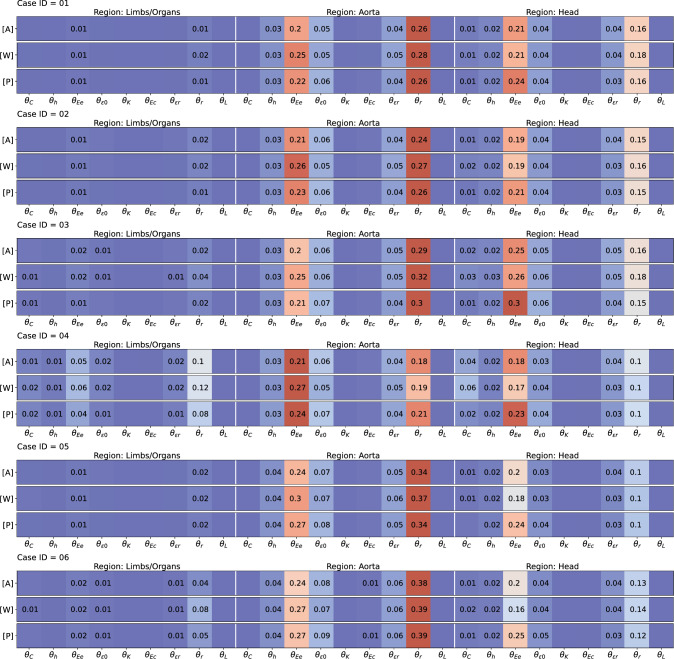
Total Sobol indices for the sensitivity of each model parameter on the cost function for patient IDs 01 to 06. At each panel, total Sobol indices are computed considering [A] ADAN, [W] Williamson, and [P] Pahlevan ejection waveforms (see Fig. 2)

**Fig. 5:**
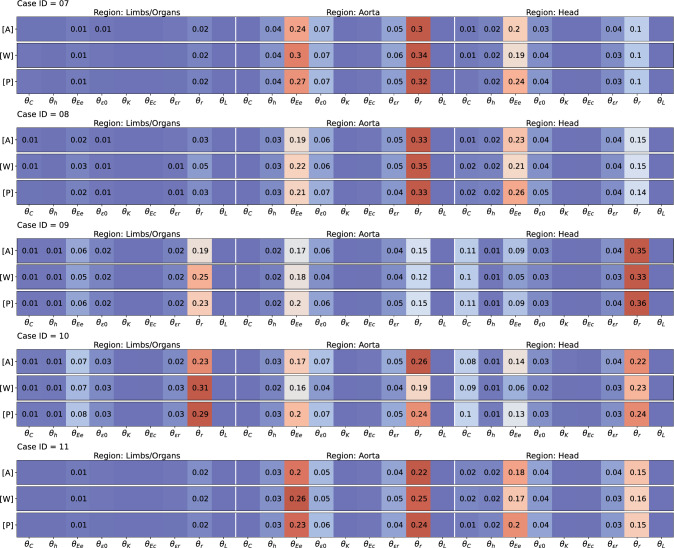
Total Sobol indices for the sensitivity of each model parameter on the cost function for patient IDs 07 to 11. At each panel, total Sobol indices are computed considering [A] ADAN, [W] Williamson, and [P] Pahlevan ejection waveforms (see Fig. 2)

The influence of each model parameter (surrogated to its multiplicative factor) on the cost function is shown in Figs. 4 and 5. Each case is labeled with the corresponding patient ID, indicating the patient data used to define the cost function. In each panel, the total Sobol indices are presented and color-coded from blue (low values) to red (high values), with empty cells denoting parameters that have index values lower than 0.01. For each patient, Sobol indices were computed for three different cardiac ejection waveforms: the first row ([A]) corresponds to the baseline waveform of the ADAN-86 model (Blanco et al. 2015); the second row ([W]) to the waveform reported in (Williamson et al. 2024); and the third row ([P]) to the waveform from (Pahlevan and Gharib 2014). These waveforms are also shown in Fig. 2.

The SA indicates that, regardless of the ejection waveform, the lumen radius ($\theta _r$) and the elastin modulus ($\theta _{Ee}$) consistently have a dominant influence across all patients. The collagen activation parameters ($\theta _{\varepsilon 0}$, $\theta _{\varepsilon r}$), compliance ($\theta _C$) and arterial wall thickness ($\theta _h$) show a secondary level of importance. Parameters with Sobol indices lower than 0.01 were considered to have a negligible effect. In most cases, the major influence comes from the aorta and head regions, being the variation in the radius and elastin of vessels in these regions responsible for more than $85\%$ of the variability in the cost function. This is consistent with the fact that the cost function depends directly on the waveforms within these regions. The patients labeled as case ID 04, 09, 10 differ from this rule, as they place a high importance on the parameters defined in the limbs/organ region, with the radius variation of this region being responsible for 10 to $40\%$ of the variability in the cost function. This issue could be explained by the presence of strong wave reflections arriving from the peripheral regions and affecting the aortic and carotid waveforms, as shown in Fig. 3. Furthermore, it is also worth highlighting that these patients are characterized by a high SYNTAX index (see Table 4), which could also suggest that these patients, who feature a higher degree of coronary artery disease, also feature more sensitivity to the parameters from the peripheral region. As it will be discussed in Sect. 5, further studies are needed to establish a concrete relation between any functional index and the sensitivity presented in this section.

**Fig. 6:**
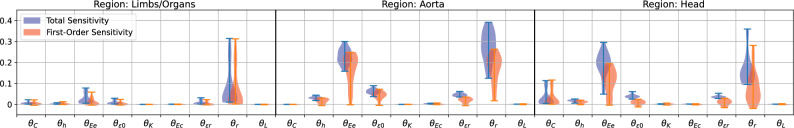
Distribution of the total and first-order Sobol indices for each parameter in the 1D blood flow model

Figure 6 shows the distribution of the total (in blue) and first-order (in red) Sobol indices, computed using SA results for all patients and different cardiac ejection waveforms. Overall, we can clearly identify three groups of parameters according to their impact on the cost function variability:

i. parameters with a high influence: the arterial radius $\theta _r$ and the elastin component $\theta _{Ee}$;
ii. parameters with mild influence: the collagen fiber distribution parameters $\theta _{\varepsilon 0}, \theta _{\varepsilon r}$, the arterial wall thickness $\theta _h$, and the terminal compliance $\theta _C$; and
iii. parameters with no/negligible influence: the viscoelastic behavior $\theta _K$, collagen component $\theta _{Ec}$, and the arterial length $\theta _L$.

## Model personalization

### Evolution strategy for numerical optimization

The path towards the personalization of 1D blood flow models to specific subjects amounts to seeking for the optimal multiplicative factor $\boldsymbol{\theta }\in {\mathbb {R}}^n$ that minimizes the discrepancy between the model prediction and the measured data. To efficiently explore the parameter space, we have turned to evolution optimization strategies, that do not require the computation of the cost function gradient (Beyer and Schwefel 2002). As for many other optimization methods, evolutionary strategies begin with an initial parameter guess $\boldsymbol{\theta }_0$, called parent, and then generate, in a stochastic manner, new parameter candidates called offsprings. For each generation, the cost function associated with the offsprings is evaluated, and these candidates are then selected based on their fitness (i.e., the value of the cost function). At the end of the optimization process, only the best individual is kept. In this work, we have chosen to use the Covariance Matrix Adaptation Evolutionary Strategy (CMA-ES) algorithm, an evolutionary strategy with an auto-adaptive covariance matrix that is learned during the optimization procedure (Hansen et al. 2003; Hansen 2006).

In its more fundamental form, the CMA-ES samples $\lambda \ge 2$ candidate solutions from a multivariate normal distribution ${\mathcal {N}}(\boldsymbol{\theta }_t, \sigma _t^2 {\boldsymbol{C}}_t)$ where the mean $\boldsymbol{\theta }_t \in {\mathbb {R}}^n$ is the incumbent solution, $\sigma _t$ is a scalar referred to as step-size and ${\boldsymbol{C}}_t \in {\mathbb {R}}^{n\times n}$ is a positive definite covariance matrix. The subindex *t* stands for the generation. The candidate solutions $\{\boldsymbol{\theta }_{t+1}^i\}_{i = 1, \ldots ,\lambda }$ are ranked from the best to the worst, in the sense of the cost function to be minimized, and then are used to update the mean as follows

12 $$\begin{aligned} \boldsymbol{\theta }_{t+1} = \boldsymbol{\theta }_{t} + c_m\sum _{i = 1}^\mu w_i (\boldsymbol{\theta }_{t+1}^{i} - \boldsymbol{\theta }_t), \qquad \text {with}\,w_1 \ge w_2 \ge \ldots \ge w_\mu \ge 0, \end{aligned}$$

where $\mu = \lfloor \lambda /2\rfloor$ defines the size of the set of best candidates, $c_m$ is a learning rate and $w_i = \ln ((\lambda +1)/2) - \ln (i)$, for each $1\le i\le \mu$. The covariance matrix update follows the rule

13 $$\begin{aligned} {\boldsymbol{C}}_{t+1} = \left( 1 - c_1 - c_\mu \sum _{i = 1}^\lambda w_i \right) {\boldsymbol{C}}_t + c_1 {\boldsymbol{p}}_{t+1}\otimes {\boldsymbol{p}}_{t+1} + c_\mu \sum _{i = 1}^\lambda w_i {\boldsymbol{z}}^i \otimes {\boldsymbol{z}}^i, \end{aligned}$$

where $c_1\in (0,1)$, $c_\mu \in (0,1)$, ${\boldsymbol{z}}_i = (\boldsymbol{\theta }_{t+1}^i - \boldsymbol{\theta }_t)/\sigma _t$ and ${\boldsymbol{p}}_{t+1}\in {\mathbb {R}}^n$ is the so-called evolution path, which starts with ${\boldsymbol{p}}_0 = {\boldsymbol{0}}$ and evolves using the rule

14 $$\begin{aligned} {\boldsymbol{p}}_{t+1} = ( 1 - n^{-1/2} ){\boldsymbol{p}}_t + \delta _p \dfrac{\boldsymbol{\theta }_{t+1} - \boldsymbol{\theta }_t}{c_m \sigma _{t}}, \end{aligned}$$

with $\delta _p$ a normalization factor chosen such that ${\boldsymbol{p}}_{t+1} \sim {\mathcal {N}}({\boldsymbol{0}},{\boldsymbol{C}}_t)$. Finally, the step-size $\sigma _t$ is updated using the Cumulative Step-size Adaptation (CSA), defining the rule

15 $$\begin{aligned} \ln \sigma _{t+1} = \ln \sigma _t + \dfrac{n^{-1/2}}{d_\sigma }\left( \dfrac{\left\| {\boldsymbol{q}}_{t+1}\right\| }{\sqrt{n} } - 1 \right) , \end{aligned}$$

with $d_\sigma$ a damping parameter that scales the magnitude of $\sigma _t$, and ${\boldsymbol{q}}_{t+1}\in {\mathbb {R}}^n$ being the evolution path starting from ${\boldsymbol{q}}_0 = {\boldsymbol{0}}$ and following the rule

16 $$\begin{aligned} {\boldsymbol{q}}_{t+1} = ( 1 - n^{-1/2} ){\boldsymbol{q}}_t + \delta _q {\boldsymbol{C}}_t^{-1/2}\dfrac{\boldsymbol{\theta }_{t+1} - \boldsymbol{\theta }_t}{c_m \sigma _{t}}, \end{aligned}$$

with $\delta _q$ a normalization factor. For further details on the CMA-ES rationale and extensions, the reader is referred to (Hansen et al. 2003; Hansen 2016; Varelas et al. 2018). The CMA-ES is a stochastic derivative-free optimization algorithm, commonly employed for black-box optimization problems and its implementation is available in the cma python library (Hansen et al. 2019). Regarding the computational burden of the CMA-ES optimization, this directly depends on the number of candidate solutions ($\lambda$) and the total number of generations. While the first one follows the rule of thumb $\lambda = \lceil 4 + 3\ln (n) \rceil$ (Hansen and Ostermeier 2001), the second is problem-dependent and is chosen such that it ensures the convergence of the optimized solution.

In the previous section, the SA ranked the model parameters according to their influence on the personalization of the 1D blood flow model based on the patient data. Hence, the SA can be understood as a strategy to reduce the parameter space dimension, focusing on optimizing only the more influential parameters. Although this approach could drastically reduce the dimension of the parameter space, and therefore the computational burden, to corroborate the usefulness of the SA results for the present context, we first address the optimization process considering the complete parameter space (with $n = 40$) and, second, we take into consideration the most influential parameters in the optimization process.

### Full-parameter space optimization

The CMA-ES algorithm was employed to estimate the optimal multiplicative factor $\boldsymbol{\theta }\in {\mathbb {R}}^{40}$, comprising ten parameters per vascular region and ten parameters for the cardiac ejection waveform. The range for each multiplicative factor is as reported in Table 3. To ensure that the optimization procedure returns physiologically plausible regimes, we constrain each parameter within its initial range. For each patient dataset, the number of offsprings is set to $\lambda = \lceil 4 + 3\ln (40)\rceil = 16$ and optimization is performed over 160 generations, which was checked sufficient to ensure convergence up to $1\%$ for each model parameter. Due to the stochastic nature of the evolutionary strategy, 40 independent optimization runs were executed per patient case to assess the robustness of the identified parameters. Each optimization run starts with different initial parameter guess. As in the SA section, the evaluation of the cost function for each offspring was conducted using a time step of $\Delta t = 0.001$ s, a heart rate of 60 bpm, and 10 cardiac cycles to ensure the cycle-to-cycle convergence. Since offspring evaluations are independent, after sampling, the population of each generation was run in parallel, resulting in an average generation time of 860 s. The total runtime for a single optimization process was approximately 38 h.

**Fig. 7:**
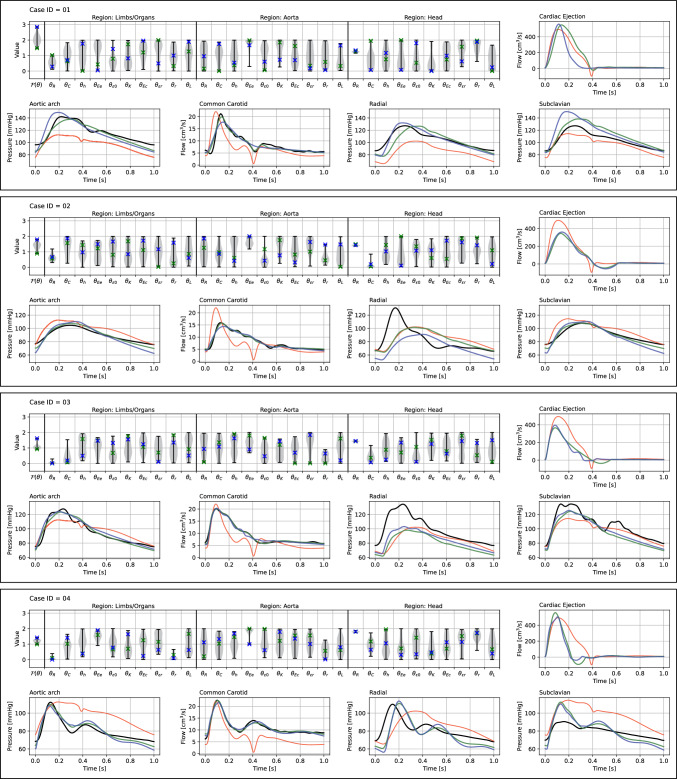
Parameter distribution and predicted waveforms for subjects 01 to 04. At each panel, baseline model (red), best (green), and worst (blue) optimized models, in terms of the cost function value, are compared to the measured data (black)

**Fig. 8:**
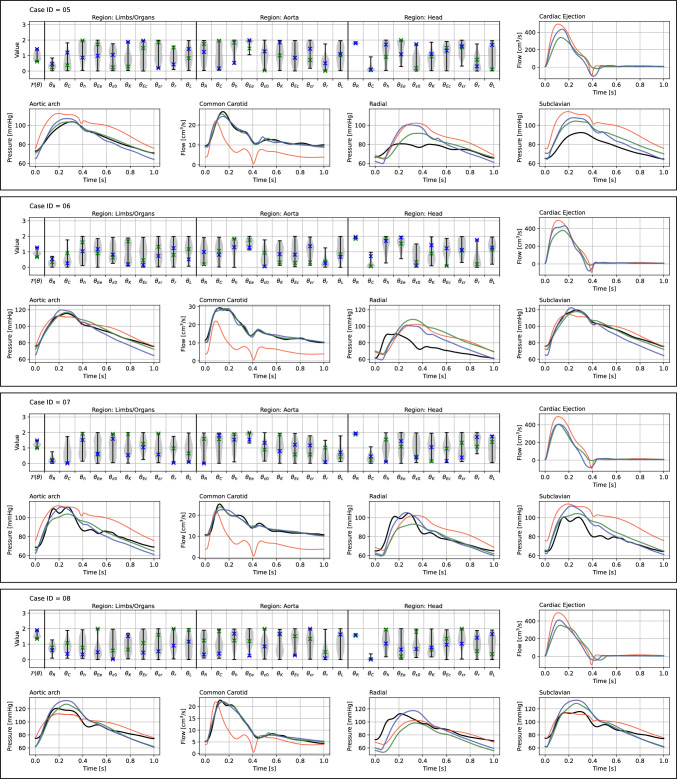
Parameter distribution and predicted waveforms for subjects 05 to 08. At each panel, baseline model (red), best (green), and worst (blue) optimized models, in terms of the cost function value, are compared to the measured data (black)

**Fig. 9:**
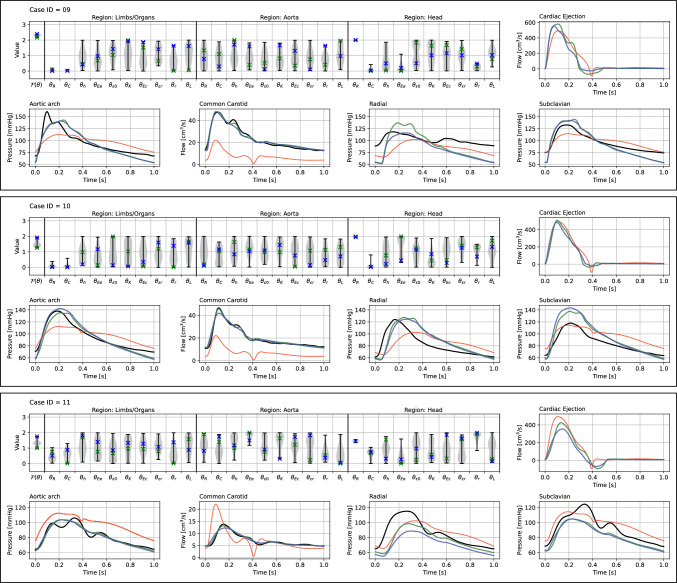
Parameter distribution and predicted waveforms for subjects 09 to 11. At each panel, baseline model (red), best (green), and worst (blue) optimized models, in terms of the cost function value, are compared to the measured data (black)

The results of the 40 optimization runs for each patient dataset are presented in Figs. 7, 8 and 9. Each panel, identified by the corresponding patient ID number, displays a violin plot showing the distribution of optimized parameters alongside a comparison between the patient’s measured waveforms and model predictions. In the violin plot, the range of the 40 estimated optimal parameters is depicted by linearly scaling each multiplicative factor to the interval [0, 2] for easier comparison, as well as the best (green cross) and worst (blue cross) parameter combinations (out of the 40 realizations), according to cost function values. The distribution of the multiplicative parameters exhibits a wide range of combinations, spanning quite uniformly the entire admissible range (as $\theta _R$ or $\theta _{Ec}$ in case ID = 01) or highlighting regions of higher probability (as $\theta _K$ in case ID = 01). This behavior suggests compensatory effects among parameters, as well as the existence of multiple local minima in the cost function, as defined in Equation (6). Some parameters, however, appear to be more narrowly determined. For instance, the terminal resistance ($\theta _R$) in the head and limbs/organ regions, and the arterial wall elastin ($\theta _{Ee}$) in the aorta, tend to converge toward specific nominal values, consistently with the relevance established by the SA findings. Similar convergence can also be observed in other parameters, such as the arterial radius in the aorta ($\theta _r$) for cases ID = 02, 03, and 06, or the same parameter in the head region for cases ID = 01, 04, 09, and 11.

In the same figures, a direct comparison between the measured waveforms and the predictions from the optimized models is also presented. Within each panel, measured data are shown in solid black; model predictions corresponding to the best and worst parameter sets (out of the 40 realizations) are shown in green and blue, respectively. Additionally, the baseline model prediction is displayed in red for reference. The ability of the optimization procedure to guide the model predicted waveforms to mimic the aortic and carotid measurements is clear in all the cases, being the optimized model able to reproduce patient-specific mean carotid flow (as in cases ID = 06, 07, and 09); ranges of aortic pressure pulse (ID = 01, 05, 07, and 09); and important deviations in the wave morphology (cases ID = 04, 07, 09, and 11) when compared to baseline model’s output signals. As stated in Sect. 2, although pressure data in the radial and subclavian arteries were also available, the cost function does not incorporate them into the optimization procedure. This, to avoid any possible bias due to unbalanced information (more pressure signals than flow measurements) and also to assess the generalization of the model predictions over vascular regions not included in the cost function. To explore this last point, the cardiac ejection and pressure waveforms in the radial and subclavian arteries are also presented for each patient. The predicted waveforms corroborate that the optimized model predictions remain physiologically consistent, considering clinically acceptable ranges and wave morphology.

**Fig. 10:**
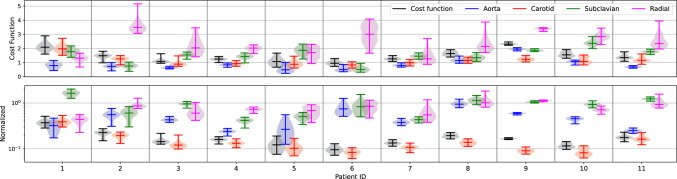
Cost function distribution (in black) and its contributions given by the aortic pressure mismatch (in blue) and carotid flow (in red) mismatch. Also, the mismatch distributions in the radial and subclavian pressure prediction, signals not included in the optimization process, are presented. In the superior panel, the distribution of cost function values is displayed. In the bottom panel, the normalized cost regarding the mismatch between the measurements and the baseline model prediction is presented

For a better comparison, the cost function distribution per patient is depicted in Fig. 10. In the superior panel, we present the distribution of the cost function across the 40 realizations (in black), together with the distribution of the specific contributions given by the aortic pressure term (in blue) and the carotid flow (in red) term to the cost function. The bottom panel shows the same distributions, following the same color code, but normalized by the discrepancy between the measurements and the baseline model predictions. In addition to the two terms that compose the cost function, we also report the error distribution of the available measurements that are not included in the optimization process. Specifically, this corresponds to the mismatch distribution for the subclavian pressure signal (shown in green) and for the radial pressure signal (shown in magenta). Concerning the value reached by the cost function, the patient cases with the best agreement between model predictions and measurements are those labeled as ID = 02, 03, 04, 05, 06, 07 and 11. For these cases, represented by the green curves in each panel of Figs. 7, 8 and 9, the predicted flow waveform closely overlaps the measured data. In cases 02 and 03, the best solution accurately reproduces the higher-order frequencies during the deceleration phase of the carotid flow and also captures the pressure scale, matching the mean pressure, though with slightly lower predictive accuracy compared to the flow. For cases 04, 05, and 06, the optimized model satisfactorily predicts both pressure and flow, with particularly notable performance in case 04, where patient data strongly deviates from the baseline predictions. In case 11, the main discrepancies are observed in the systolic phase of the pressure waveform, where the optimized model tends to smooth out the pressure surge present in late systole. In turn, the least accurate results correspond to the blue curves in cases ID = 01, 09, and 10. In these cases, larger discrepancies are observed between the model predictions and the measurements, particularly in the aortic pressure signal. Referring back to the sensitivity analysis presented in the previous section, it is worth noting that cases 09 and 10 are the ones in which parameters from the limbs/organ region play a considerable role regarding the cost function. This may indicate either pathological conditions or anatomical variations not accounted for in the present model. Finally, it is remarkable that, for all the patients, the worst scenarios in terms of the normalized cost function have values lower than the unit. This means that, even taking into account the stochastic nature of the optimization procedure, the resulting models are notably closer than the baseline model to the patient measurements. Furthermore, in all the cases, but for the ID = 01, the optimization procedure matches more accurately the flow signal than the pressure signal.

The model’s ability to reproduce waveforms in vascular regions not directly involved in the optimization procedure can be assessed through the mismatch distributions reported in Fig. 10. For most subjects, except for cases ID = 01 and 05, the mismatch in the subclavian artery is lower than that observed in the radial artery. This difference can be attributed to the relative location of these vessels: the subclavian artery lies between two calibrated regions (the aorta and the carotid artery), whereas the radial artery is located further downstream, and discrepancies may accumulate along this segment without being directly corrected during the optimization process. This observation is further supported by direct comparisons between model predictions and measured signals in Figs. 7, 8 and 9. Rather than focusing solely on predictive accuracy (which may be misleading since these signals are not included in the calibration), it is important to emphasize that, across all patients, the predicted radial and subclavian waveforms remain physiologically consistent, exhibiting realistic morphologies and pressure ranges within clinically acceptable limits. A similar observation holds for the predicted cardiac ejection profiles. The proposed ten-parameter parametrization strategy provides sufficient flexibility to capture variations in cardiac output, ejection duration, and, most importantly, waveform morphology, which strongly influences global hemodynamics, while avoiding spurious or non-physiological behavior.

### Reduced-parameter space optimization

To evaluate the influence of parameters with different sensitivities in the optimization procedure, two scenarios are considered: (i) when only the parameters related to the boundary conditions are optimized; and (ii) when, in addition to (i), also the parameters identified as highly influential in the sensitivity analysis are optimized.

**Fig. 11:**
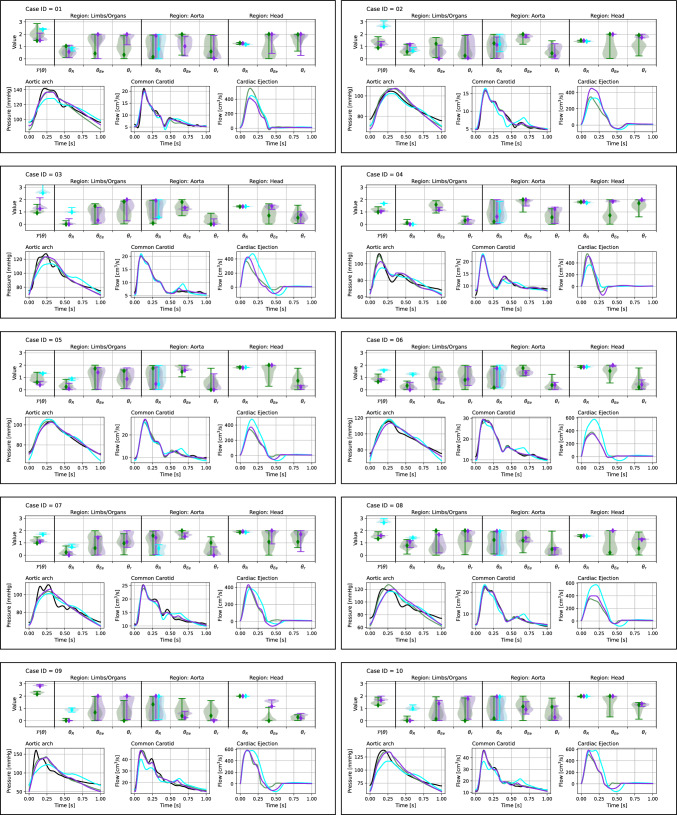
Optimized model predictions for each patient-specific data (curves in black). In each panel, the green line stands for the predictions using the full-parameter optimized model, in cyan the predictions considering only the boundary conditions parameters, and in purple for the model calibrated with the most influential parameters according to the SA

In the first case, the multiplicative factors $\boldsymbol{\theta }\in {\mathbb {R}}^{40}$ are restricted to the resistance and cardiac waveform parameters, as listed in Table 3, while all other factors are fixed to $\theta = 1$, thereby preserving the nominal baseline values for the geometric and mechanical parameters. For each patient, the number of parameters to be identified is therefore $n = 13$, the offspring size is set to $\lambda = \lceil 4 + 3\ln (13)\rceil = 12$, and the optimization is carried out over 160 generations, with 40 independent identification executions performed from different sampling seeds. The resulting distributions of the resulting resistance parameters, along with the aortic arch pressure, common carotid flow, and cardiac ejection waveforms, are shown in cyan in Fig. 11. The results reveal that, when the remaining parameters are fixed, the terminal resistances converge close to a common value with minimal sensitivity to the stochastic nature of the evolutionary algorithm. For the aorta group, the resistance turns out to be irrelevant in the optimization procedure. As previously detailed, this is because this region was defined without terminal vessels, and so the resistance does not play any effective role in the model. Comparing the model predictions in cyan (boundary-condition-only optimization) against the patient data in black and the full-parameter predictions (from the previous section) in green highlights the role of boundary conditions in determining the overall waveform shape and mean values. However, the optimization using such a reduced parameter space is insufficient to capture finer waveform details, such as high-frequency flow oscillations in patient ID = 07, peak flow values in patient ID = 09, or the systolic pressure morphology in cases ID = 01 and 03. This suggests that while boundary conditions strongly influence global hemodynamic behavior, accurate personalization of local waveform features requires the inclusion of mechanical and geometric parameters in the optimization.

In the second case, the parameters identified as highly influential in the sensitivity analysis are incorporated into the identification procedure. Specifically, the multiplicative factors related to collagen fiber distribution ($\theta _{\varepsilon 0}, \theta _{\varepsilon r}$), arterial wall thickness ($\theta _h$), terminal compliance ($\theta _C$), viscoelastic behavior ($\theta _K$), collagen stiffness ($\theta _{Ec}$) and arterial length ($\theta _L$) are fixed to 1, thereby preserving their nominal baseline values. The parameters to be identified in this scenario consist of the 13 boundary-condition parameters, along with the arterial radius and elastin component parameters for each vascular region. Thus, the total number of parameters becomes $n = 19$. The offspring size is set to $\lambda = \lceil 4 + 3\ln (19)\rceil = 13$, and the optimization is carried out over 160 generations, with 40 independent processes performed. The resulting distributions of the resistance parameters, together with the aortic arch pressure, common carotid flow, and cardiac ejection waveforms, are shown in purple in Fig. 11. The results confirm the findings from the global sensitivity analysis section, in that the predictions obtained with the optimized model employing the reduced-parameter space (purple curves) closely match those obtained with the full-parameter space (green curves). The main discrepancies between the two cases are observed in the cardiac ejection waveform, suggesting the presence of local minima in the cost function. These differences between these cardiac waveforms appear to diminish as we move towards more distal regions in the cardiovascular system, such that by the time the flow reaches the common carotid artery, the variability is largely reduced. In addition, the variability in the distribution of the resulting parameters is reduced when we remove parameters with low sensitivity (purple violin plots are more narrow than green ones), showing that the optimization including all parameters is not only unnecessary, but may mislead the characterization of the most influential parameters.

A direct comparison of the optimization capabilities among these three cases (full parameter space, boundary-condition-only parameters, and reduced parameter space) is presented in Table 5, together with the individual matching of each waveform involved in the optimization (aortic pressure and carotid flow), as well as those not included in the process (subclavian and radial pressures). Overall, the case when only the parameters that define the boundary conditions (Only-BC case) shows a worse optimization result, with a larger mean cost function, together with reduced values for the standard deviation. This is in agreement with the previous conclusions that this reduced set of parameters is more likely to converge to a specific region of the parameter space, but with clear limitations in capturing the waveforms’ details. On the other hand, when the parameters with a high sensitivity are included (Reduced-parameter case), the results are similar to or even better than those obtained in the Full-Parameter case, also featuring, in many cases, reductions in the standard deviation. Worsening the performance when more parameters are considered in the calibration procedure can be explained by the involvement of low-sensitivity parameters, which degrade the ability of the method to find effective local minima.

**Table 5:** Cost function distribution resulting from the model optimization using all the parameters (Full-parameter case), only the ones related to boundary conditions (Only-BC case), and the most sensitive ones (Reduced-parameter case)

	Cost Function			Aorta	Carotid	Subclavian	Radial
	Best	Worst	Mean ± Std	Mean ± Std	Mean ± Std	Mean ± Std	Mean ± Std
ID = 01							
Full-parameter	1.47	2.85	$2.05 \pm 0.32$	$0.77 \pm 0.17$	$2.01 \pm 0.29$	$1.77 \pm 0.2$	$1.28 \pm 0.2$
Only-BC	2.37	2.47	$2.41 \pm 0.03$	$0.86 \pm 0.01$	$2.25 \pm 0.03$	$1.56 \pm 0.1$	$1.56 \pm 0.01$
Reduced-parameter	1.5	2.09	$1.67 \pm 0.18$	$0.56 \pm 0.08$	$1.57 \pm 0.17$	$1.7 \pm 0.07$	$1.71 \pm 0.19$
ID = 02							
Full-parameter	0.89	1.79	$1.36 \pm 0.21$	$0.71 \pm 0.14$	$1.22 \pm 0.15$	$0.74 \pm 0.16$	$3.53 \pm 0.39$
Only-BC	2.6	3.08	$2.72 \pm 0.16$	$0.51 \pm 0.11$	$2.67 \pm 0.17$	$0.63 \pm 0.13$	$4.16 \pm 0.05$
Reduced-parameter	1.11	1.41	$1.23 \pm 0.09$	$0.75 \pm 0.04$	$0.97 \pm 0.1$	$0.65 \pm 0.05$	$4.09 \pm 0.43$
ID = 03							
Full-parameter	0.92	1.62	$1.07 \pm 0.15$	$0.63 \pm 0.05$	$0.91 \pm 0.16$	$1.54 \pm 0.1$	$2.18 \pm 0.54$
Only-BC	2.54	3.31	$2.62 \pm 0.22$	$1.24 \pm 0.06$	$2.3 \pm 0.22$	$1.73 \pm 0.06$	$3.36 \pm 0.12$
Reduced-parameter	1.26	2.15	$1.36 \pm 0.25$	$0.88 \pm 0.13$	$1.04 \pm 0.21$	$1.69 \pm 0.02$	$2.8 \pm 0.39$
ID = 04							
Full-parameter	0.99	1.42	$1.2 \pm 0.1$	$0.83 \pm 0.07$	$0.92 \pm 0.09$	$1.41 \pm 0.14$	$1.99 \pm 0.16$
Only-BC	1.68	1.71	$1.69 \pm 0.01$	$1.14 \pm 0.01$	$1.25 \pm 0.01$	$0.68 \pm 0.01$	$2.41 \pm 0.01$
Reduced-parameter	1.03	1.13	$1.08 \pm 0.03$	$0.87 \pm 0.03$	$0.63 \pm 0.02$	$1.16 \pm 0.05$	$2.1 \pm 0.09$
ID = 05							
Full-parameter	0.61	1.42	$0.96 \pm 0.23$	$0.52 \pm 0.22$	$0.91 \pm 0.21$	$1.84 \pm 0.25$	$1.7 \pm 0.39$
Only-BC	1.32	1.38	$1.33 \pm 0.02$	$0.52 \pm 0.02$	$1.22 \pm 0.03$	$1.97 \pm 0.04$	$1.97 \pm 0.02$
Reduced-parameter	0.39	0.7	$0.56 \pm 0.11$	$0.21 \pm 0.04$	$0.52 \pm 0.12$	$1.72 \pm 0.06$	$1.55 \pm 0.36$
ID = 06							
Full-parameter	0.66	1.28	$0.93 \pm 0.15$	$0.52 \pm 0.13$	$0.8 \pm 0.11$	$0.52 \pm 0.15$	$3.01 \pm 0.65$
Only-BC	1.55	1.67	$1.61 \pm 0.05$	$0.59 \pm 0.01$	$1.5 \pm 0.05$	$0.57 \pm 0.03$	$3.54 \pm 0.12$
Reduced-parameter	0.76	0.89	$0.8 \pm 0.03$	$0.49 \pm 0.02$	$0.63 \pm 0.05$	$0.54 \pm 0.03$	$3.52 \pm 0.55$
ID = 07							
Full-parameter	0.98	1.49	$1.21 \pm 0.1$	$0.79 \pm 0.08$	$0.97 \pm 0.11$	$1.45 \pm 0.1$	$1.39 \pm 0.41$
Only-BC	1.68	1.81	$1.72 \pm 0.04$	$0.94 \pm 0.02$	$1.44 \pm 0.04$	$1.36 \pm 0.04$	$2.22 \pm 0.05$
Reduced-parameter	1.08	1.24	$1.13 \pm 0.05$	$0.75 \pm 0.04$	$0.84 \pm 0.07$	$1.44 \pm 0.08$	$1.63 \pm 0.41$
ID = 08							
Full-parameter	1.34	1.91	$1.59 \pm 0.15$	$1.15 \pm 0.11$	$1.16 \pm 0.14$	$1.33 \pm 0.16$	$2.28 \pm 0.47$
Only-BC	2.64	2.99	$2.76 \pm 0.11$	$1.16 \pm 0.03$	$2.5 \pm 0.13$	$1.16 \pm 0.03$	$2.99 \pm 0.07$
Reduced-parameter	1.58	1.61	$1.6 \pm 0.01$	$1.38 \pm 0.01$	$0.81 \pm 0.02$	$1.42 \pm 0.01$	$2.95 \pm 0.37$
ID = 09							
Full-parameter	2.16	2.39	$2.28 \pm 0.06$	$1.95 \pm 0.05$	$1.23 \pm 0.11$	$1.87 \pm 0.06$	$3.36 \pm 0.08$
Only-BC	4.52	5.09	$4.7 \pm 0.17$	$2.77 \pm 0.05$	$3.79 \pm 0.2$	$1.23 \pm 0.06$	$3.07 \pm 0.16$
Reduced-parameter	2.82	2.93	$2.88 \pm 0.03$	$2.51 \pm 0.06$	$1.4 \pm 0.07$	$1.84 \pm 0.06$	$3.86 \pm 0.19$
ID = 10							
Full-parameter	1.26	1.92	$1.53 \pm 0.19$	$1.05 \pm 0.08$	$1.17 \pm 0.22$	$2.35 \pm 0.17$	$2.86 \pm 0.23$
Only-BC	3.68	4.1	$3.79 \pm 0.11$	$1.88 \pm 0.06$	$3.29 \pm 0.1$	$1.46 \pm 0.11$	$4.13 \pm 0.07$
Reduced-parameter	1.66	2.0	$1.74 \pm 0.09$	$1.36 \pm 0.07$	$1.08 \pm 0.1$	$2.24 \pm 0.08$	$3.52 \pm 0.28$
ID = 11							
Full-parameter	1.0	1.75	$1.34 \pm 0.16$	$0.68 \pm 0.05$	$1.21 \pm 0.16$	$1.75 \pm 0.08$	$2.49 \pm 0.42$
Only-BC	2.57	2.86	$2.71 \pm 0.1$	$1.06 \pm 0.04$	$2.49 \pm 0.11$	$1.86 \pm 0.1$	$3.2 \pm 0.16$
Reduced-parameter	1.46	1.79	$1.63 \pm 0.1$	$0.86 \pm 0.06$	$1.38 \pm 0.12$	$1.58 \pm 0.11$	$3.0 \pm 0.48$

For each patient case, the mean and standard deviation of the cost function and the pressure and flow components are presented

### Influence on hemodynamic biomarkers

**Fig. 12:**
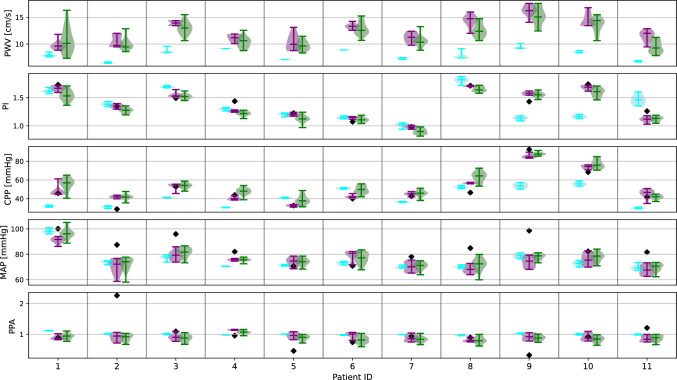
Variability of hemodynamic biomarkers across the 40 optimized models for each parameter set. From top to bottom: Carotid-Femoral Pulse Wave Velocity (PWV), Carotid Pulsatility Index (PI), Aortic Central Pulse Pressure (CPP), Radial Artery Mean Arterial Pressure (MAP), and Radial Artery Pulse Pressure Amplification (PPA). For each patient, each violin plot corresponds to the prediction using the optimized model with the full parameter set (in green), with the boundary-condition set (in cyan), and with the reduced parameter space according to the SA (in purple). Black markers stand for the biomarker value computed from the patient measurements

As a complementary evaluation of the individualized models, five common hemodynamic biomarkers were assessed: carotid–femoral pulse wave velocity, carotid pulsatility index, central pulse pressure, radial mean arterial pressure, and radial artery pulse pressure amplification. For each patient, and for each parameter subset considered in the optimization strategy (full parameter space, boundary-condition-only parameters, and reduced parameter space), the variability across the 40 optimized models is displayed in Fig. 12.

It is worth noting that both the carotid pulsatility index (PI) and the central pulse pressure (CPP) can be directly derived from the carotid and aortic signals used in the optimization procedure. This makes them particularly suitable for a straightforward comparison between measured data and model predictions. The PI, defined as the difference between peak systolic and end-diastolic carotid flow normalized by the mean flow, is consistently predicted with low variability across all patients. Among the different strategies, optimization using the full parameter set tends to display slightly higher variability. This behavior can be explained by the broader dispersion of optimized parameters when more degrees of freedom are allowed in the calibration. Despite this, the agreement between model-predicted PI and measured PI is generally good, with particularly accurate matches for patients ID = 01, 02, 03, 05, 07, 08, and 09. Similarly, the model-based CPP had a mean value close to the measured-based CPP, in almost all the cases. Furthermore, in this case, the optimization with the reduced set of parameters produces a lower variability than the full parameter set, while preserving a nearly identical mean value. This trend reinforces the conclusions of the sensitivity analysis and the improvement in the search for local minima by reducing the degrees of freedom, see Fig. 11. Finally, although the variability is the smallest when only boundary condition parameters are optimized, these cases also yield the largest deviations from the measured CPP. This emphasizes that boundary conditions alone are insufficient to capture patient-specific dynamics; local vessel parameters play a crucial role, alongside outlet calibration, in ensuring accurate predictions of blood-flow-derived biomarkers.

For the carotid–femoral pulse wave velocity (PWV), defined as the ratio between the distance along the arterial pathway and the foot-to-foot time delay of pressure waveforms, no direct patient-specific reference values could be obtained due to the absence of femoral pressure recordings. Nonetheless, the model predictions are highly consistent, with the mean values obtained with the full and reduced parameter sets very close to each other across the 40 optimization runs. Furthermore, although no direct PWV measurements are available, predicted values are in the expected range between 8 and 12 m/s reported in the literature for similar cohorts (hypertensive patients and a mean age of $60 \pm 8$ years), as in Diaz et al. (2018); de Mendonça et al. (2018). This consistency likely reflects that even with limited patient-specific information available in the limbs/organ region, it is possible to gain insight about hemodynamics at peripheral locations and their impact on clinical biomarkers. A comparable trend is also observed in Fig. 11, where parameters associated with the limbs/organ region show a higher variability than in the other regions. Turning to peripheral biomarkers, the radial mean arterial pressure (MAP) and the radial artery pulse pressure amplification (PPA) provide an opportunity to assess the reliability of the model predictions in regions where no direct data were incorporated into the optimization process. The predictions for both biomarkers remain very consistent regardless of the parameter set considered. This strongly suggests that the blood flow dynamics in these non-monitored regions is primarily driven by global circulatory conditions, determined by the cardiac ejection waveform, terminal resistances, and central vessel properties, while local variations in vascular mechanical and geometrical parameters play only a secondary role. The discrepancies with respect to the measured data can be explained by the fact that these signals were not included in the optimization procedure. In addition, note that synchronization of respiratory phases was not considered, which can explain the discrepancies in the radial artery mean pressure values.

## Discussion

In recent years, the pursuit of individualized models in computational hemodynamics has become a crucial step toward translational research and the integration of such models into clinical practice. Concerning the 1D theory of blood flow, this integration is still in its early stages. In this work, we addressed a key aspect of this challenge: the integration of patient-specific pressure and flow waveforms into a parameter identification framework to address the calibration of model parameters that characterize the systemic circulation. By coupling the ADAN-86 model with the Covariance Matrix Adaptation Evolutionary Strategy (CMA-ES), we sought to optimize mechanical and geometric parameters across different vascular regions, as well as parameters that define the inflow and outlet boundary conditions, tackling the inherent difficulties of a high-dimensional, small-data problem, and illustrating the effectiveness of the approach with a set of real-world data.

In contrast to previous studies, the present work explicitly integrates a comprehensive global sensitivity analysis as a preliminary step to guide the calibration process. Existing approaches based on data assimilation, such as reduced-order Unscented Kalman Filter methods (Caiazzo et al. 2017; Müller et al. 2019; Zhang et al. 2020), have demonstrated efficient parameter estimation but typically rely on reduced or structured parameter spaces and sequential updates. Similarly, flexible estimation frameworks (Arthurs et al. 2020) focus on parameter identification under prescribed model structures. In contrast, the present work addresses a high-dimensional calibration problem and explicitly leverages sensitivity analysis to guide parameter reduction and improve interpretability. According to our findings, the reference vessel radius and the elastin stiffness parameter of the arterial wall in the aorta consistently emerged as the most influential parameters, an outcome aligned with the physiological role of the aorta as the primary determinant of systemic compliance. Furthermore, we have shown the importance of identifying the morphological parameters of cardiac ejection waveform, in line with the results also reported in Müller et al. (2019); Zhang et al. (2020). In contrast, other parameters, such as the collagen fiber response to stress, arterial wall thickness, or the compliance of Windkessel terminal elements, were found to be of secondary importance. These insights suggest the possibility of reducing the dimensionality of the parameter space, enabling a more computationally efficient model personalization with minimal or no loss of predictive accuracy. However, unlike previous works, we systematically exploit this information to reduce the effective dimensionality of the inverse problem, thereby improving robustness and interpretability. While the sensitivity patterns appear consistent across patients, larger cohorts are required to confirm these findings and to assess inter-individual variability more robustly. In addition, the current framework does not explicitly account for measurement uncertainty, which may affect both the sensitivity analysis and the calibration results. Future work will focus on incorporating uncertainty quantification and probabilistic inference methods to better characterize parameter variability and improve robustness.

Regarding the parameter identification of the hemodynamic model, our results confirmed the ability of the 1D blood flow model employed here to deliver predictions that match real-world patient data. Despite the presence of multiple local minima in the cost function and the compensatory effects among parameters, the optimization proved effective in producing subject-specific models with moderate to high accuracy, while avoiding spurious predictions in vascular regions lacking direct measurements. Similar identifiability challenges have been reported in previous studies on inverse problems in cardiovascular modeling, where parameter estimation in one-dimensional arterial networks is known to be ill-posed and prone to non-unique solutions (Lombardi 2014). In such contexts, compensatory effects between parameters can lead to multiple parameter combinations producing similar hemodynamic outputs. The present work extends these observations by providing a systematic quantification of such effects through repeated optimization runs and sensitivity-informed parameter selection. Furthermore, by considering subsets of the full parameter space, we were able to isolate the limitations of calibrating only boundary condition parameters (inflow and outlet boundary data) and also able to highlight the interference effect that parameters of secondary importance have on the estimate of parameters of greater relevance as determined by the sensitivity analysis. These findings should be understood taking into account the limited cohort, but provide substantial evidence about the strong influence of boundary conditions on hemodynamic simulations, while at the same time reinforcing the critical role of local intra-individual variability in the mechanical parameters for the fine-scale description of flow and pressure waveforms.

The ability of the model to reproduce pressure waveforms in vascular territories not included in the calibration (subclavian and radial arteries), as well as global biomarkers, represents an important validation aspect. While many studies in computational hemodynamics focus on fitting measurements at specific locations, fewer works evaluate predictive performance outside the calibration domain. The results obtained here suggest that the proposed framework is capable of characterizing global hemodynamic behavior rather than overfitting to localized measurements.

This study has several limitations that must be addressed in future work. Increasing the size of the patient population and exploring alternative similarity metrics for comparing model predictions with measurements are important key steps that deserve further exploration. In addition, incorporating a refined patient-specific geometric description acquired from medical images could further enhance the physiological realism of the model and its applicability in clinical practice, for instance, by adding data corresponding to vessel sizes acquired from ultrasound measurements in multiple peripheral vessels, or also adding patient-specific anthropometric information, such as weight, height and size of body segments for better vessel length, and size calibration. For example, the sensitivity analysis revealed that the lumen radius was a very influential parameter. A patient-specific characterization of lumen radius in certain vascular segments could help in reducing the parameter variability and compensatory effects. At last, it would also be worth exploring the incorporation of additional physiological constraints in the form of penalization terms in the loss function, in order to guide the optimization procedure to deliver predictions consistent with the domain knowledge. Examples could be the definition of specific ranges for the pulse wave velocity or the pulse pressure augmentation, among other hemodynamic biomarkers.

## Final remarks

In this work, a cohort of 11 patients with available hemodynamic measurements of pressure and flow rate waveforms was used to test a parameter identification procedure seeking to build patient-specific 1D blood flow models. Blood flow was simulated in the ADAN-86 model. The network was divided into vascular regions where parameters were identified in a differentiated manner. An evolutionary optimization strategy was applied to address the high-dimensional parameter optimization problem. To this end, we introduced multiplicative factors as surrogate model parameters to investigate, through a global sensitivity analysis, each parameter’s contribution to the discrepancy between generic model predictions and patient-specific data. This approach enabled the identification of the most influential parameters as well as those that can be safely fixed at baseline values without compromising the predictive capabilities of the model.

The optimization results showed the potential of this approach for constructing subject-specific 1D models of the circulation, yielding accurate predictions even in small-data scenarios. Although the findings suggest the presence of multiple minima, the use of the global sensitivity analysis helped mitigate this challenge by reducing the dimensionality of the parameter space and, consequently, the interactions and compensatory effects among parameters. These compensatory effects are well documented in the literature and suggest that, in practice, reducing the variability of model parameters, rather than enforcing strict uniqueness, may be a more realistic objective in the development of subject-specific models. Remarkably, the stochastic nature of the evolutionary strategy also allowed us to quantify the variability of model predictions with respect to the multiplicity of local minima.

Despite the limitations discussed, the results of this study clearly underscore the capability of 1D blood flow models, coupled to proper numerical optimization strategies, to support the development of patient-specific simulations, opening new avenues towards the effective translation of these models into medical routine.

## Data Availability

No datasets were generated or analysed during the current study.
